# Cockayne Syndrome Group B (CSB): The Regulatory Framework Governing the Multifunctional Protein and Its Plausible Role in Cancer

**DOI:** 10.3390/cells10040866

**Published:** 2021-04-10

**Authors:** Zoi Spyropoulou, Angelos Papaspyropoulos, Nefeli Lagopati, Vassilios Myrianthopoulos, Alexandros G. Georgakilas, Maria Fousteri, Athanassios Kotsinas, Vassilis G. Gorgoulis

**Affiliations:** 1 Biomedical Sciences Research Center Alexander Fleming, Institute for Fundamental Biomedical Research, 16672 Athens, Greece; spyropoulou@fleming.gr (Z.S.); fousteri@fleming.gr (M.F.); 2 Molecular Carcinogenesis Group, Department of Histology and Embryology, School of Medicine, National and Kapodistrian University of Athens, 11527 Athens, Greece; A.Papaspyropoulos@med.uoa.gr (A.P.); nlagopati@med.uoa.gr (N.L.); 3 Biomedical Research Foundation of the Academy of Athens, 11527 Athens, Greece; 4 Department of Pharmacy, National and Kapodistrian University of Athens, 15771 Athens, Greece; vmyriant@pharm.uoa.gr; 5 DNA Damage Laboratory, Physics Department, School of Applied Mathematical and Physical Sciences, National Technical University of Athens (NTUA), 15780 Athens, Greece; alexg@mail.ntua.gr; 6 Faculty of Biology, Medicine and Health, Manchester Academic Health Science Centre, University of Manchester, Manchester M20 4GJ, UK; 7 Center for New Biotechnologies and Precision Medicine, Medical School, National and Kapodistrian University of Athens, 11527 Athens, Greece

**Keywords:** Cockayne syndrome, Cockayne syndrome protein B, CSB, ERCC6, cancer, Cockayne syndrome pathologies

## Abstract

Cockayne syndrome (CS) is a DNA repair syndrome characterized by a broad spectrum of clinical manifestations such as neurodegeneration, premature aging, developmental impairment, photosensitivity and other symptoms. Mutations in Cockayne syndrome protein B (CSB) are present in the vast majority of CS patients and in other DNA repair-related pathologies. In the literature, the role of CSB in different DNA repair pathways has been highlighted, however, new CSB functions have been identified in DNA transcription, mitochondrial biology, telomere maintenance and p53 regulation. Herein, we present an overview of identified structural elements and processes that impact on CSB activity and its post-translational modifications, known to balance the different roles of the protein not only during normal conditions but most importantly in stress situations. Moreover, since CSB has been found to be overexpressed in a number of different tumors, its role in cancer is presented and possible therapeutic targeting is discussed.

## 1. Introduction

Cockayne syndrome (CS) is a rare autosomal recessive disorder characterized by progressive neurodegeneration, mental retardation, developmental abnormalities, retinal degeneration, physical impairment, severe photosensitivity and premature aging [1]. The syndrome has been mainly linked to mutations in the *ERCC8* and *ERCC6* genes encoding for Cockayne syndrome protein A (CSA) and Cockayne syndrome protein B (CSB), respectively [2,3]. The majority of patients carry mutations in the latter gene, and although CSB has been the focus of intense research, important details of the underlying mechanistic and regulatory framework are still unknown [4].

## 2. Cockayne Syndrome Protein B (CSB)

The excision repair cross-complementation group 6 (*ERCC6*) gene has been mapped to chromosome 10 and encodes for a 1493 amino acid protein (CSB) with a molecular weight of 168 kDa [3]. CSB belongs to the SWI2/SNF2 family of ATP-dependent chromatin remodelers and exhibits DNA and nucleosome-stimulated ATP hydrolytic activities [5,6,7]. Furthermore, it has been reported that CSB catalyzes the annealing of complementary single-stranded DNA molecules and possesses strand exchange activity [8]. Moreover, CSB can change the conformation of DNA by introducing negative supercoils, a process which was proposed to be dependent on ATP binding since it occurred more frequently in the presence of non-hydrolyzable ATP analogs [9].

A number of different cellular roles, recently reviewed in [4], have been attributed to CSB. Firstly, CSB protein is a major player of the transcription-coupled nucleotide excision repair (TC-NER or TCR) pathway, the subpathway of NER that removes transcription-blocking DNA lesions from the transcribed strand of active genes. Substrates for repair via the NER pathway mainly include photolesions produced by UV irradiation such as cyclobutane pyrimidine dimers (CPD) and (6,4)-pyrimidine-pyrimidone photoproducts (6-4PPs), environmental mutagens such as polycyclic aromatic hydrocarbons and bulky DNA adducts produced by chemotherapeutic agents such as cisplatin [10]. Additional structurally unrelated lesions, which are not repaired as expected by the base excision repair (BER) machinery, but are substrates of NER, include tandem base modifications such as G [8–5m]T, purine 5′,8-cyclonucleosides, interstrand cross-links and DNA–protein crosslinks [11,12]. The clinical importance of NER is evident in patients with congenital diseases and syndromes in which NER is deficient, showing symptoms of premature aging and photosensitivity, such as CS and trichothiodystrophy (TTD), increased cancer risk, such as xeroderma pigmentosum (XP), or other related pathologies. The role of CSB in TC-NER implicates RNA polymerase II (RNAPII) and together they have been implicated in the early steps of DNA damage recognition. Briefly, it has been found that while under normal conditions, CSB transiently interacts with RNAPII, this interaction is stabilized upon DNA damage [13]. In addition, CSB is required for the recruitment of the CSA/DDB1-Cul4A-RBX1 E3 ubiquitin ligase (CRL4^CSA^) in complex with the COP9 signalosome, key NER factors and chromatin modifiers such as p300 histone acetyltransferase and HMGN1 at the site of damage-stalled RNA Pol II [14]. Upon efficient repair, a role of CSB in transcription recovery from promoter proximal sites, which takes place via a CSB-mediated association of the PAF1 complex with RNAPII, has been also highlighted recently [15]. Besides the TC-NER repair pathway, CSB plays a role in the repair of oxidative DNA lesions via BER, in interstrand crosslink (ICL) repair, in DNA double-strand break (DSB) repair and checkpoint activation [16,17,18,19,20,21]. Finally, there is a number of studies indicating that CSB is involved in transcription, in chromatin remodeling, in nucleolar rDNA transcription by RNA polymerase I, in mitochondrial function, in enhancement of the p53–chromatin association, in p53 ubiquitination, in cell division completion and in telomere maintenance [22,23,24,25,26,27,28,29,30]. 

## 3. Regulatory Framework of CSB

### 3.1. Structural Regulatory Elements

**Figure 1:**
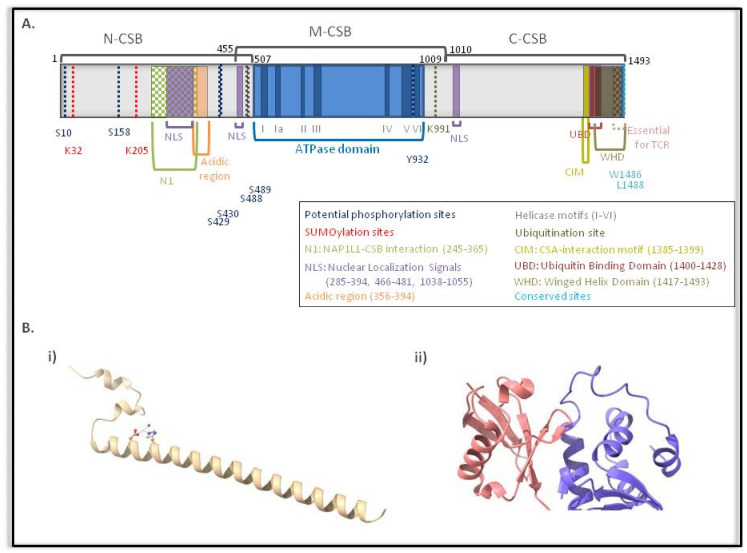
(**A**) Schematic representation of Cockayne syndrome protein B (CSB) structure. (**B**) Crystal structures of ERCC6 proteins (*i*) A ribbon representation of the N-terminal coiled coil domain of the human ERCC6 (PDB id: 4cvo; Uniprot id: q03468); (*ii*) the structure of the winged helix domain of a specific ERCC6 variant (PDB id: 6a6i; Uniprot id: q59ff6) in complex with ubiquitin (ERCC6 ribbon: blue, ubiquitin ribbon: red) [46,47].

The multiple roles of CSB highlight the need for a complex and reliable regulatory mechanism to control protein function under normal conditions, and more importantly under genotoxic stress conditions. The CSB protein can be divided into three distinct segments: the N-terminal, the C-terminal and the central ATPase domain consisting of seven conserved helicase motifs (Figure 1). At the N-terminal, an acidic-rich region has been defined, whereas at the C-terminal, a ubiquitin-binding domain (UBD) and a winged helix domain (WHD) have been identified at amino acid residues 1400–1428 and 1417–1493, respectively [19,31,32]. An evolutionarily conserved CSA interaction motif (CIM) located between amino acids 1385–1399 has also been discovered recently [33]. Two nuclear localization sequences (NLSs) have been found on either side of the ATPase domain (amino acid residues 466–481 and 1038–1055), whereas a third NLS has been predicted via computational analysis at amino acid residues 285–354 [34].

Mutational studies and genetic analyses of the mutations carried by CS patients have revealed the functional importance of the different CSB domains (Figure 1). Cho et al. has shown that removal of the 245–365 amino acids located at the N-terminus of the protein disrupted the interaction between CSB and the NAP1L1 histone chaperone, affecting the ATP-dependent chromatin remodeling activity of CSB [35]. Interestingly, apart from the UBD that is considered essential for TC-NER [28], the last 30 C-terminal amino acid residues (1464–1493) have been identified as essential for the repair of UV-induced DNA lesions by TC-NER, given that the interaction of CSB with RNAPII and chromatin after UV radiation, as well as the translocation of CSA to the nuclear matrix, were found to be affected in mutants carrying such a deletion (CSB1−1463) [36]. These C-terminal amino acids include the conserved W1486 and L1488 residues, which are part of the hydrophobic core of the identified WHD [19]. The WHD of CSB, independently of its ubiquitin-binding activity, was found to be essential for RNAPII abundance regulation at promoter proximal pause (PPP) sites of actively transcribed genes [37]. On the other hand, both the WHD and the UBD are considered necessary for the interaction of the C-terminal part with the CSB ATPase domain, an interaction, which is disrupted upon UV-induced damage via proper folding of the WHD [38]. Finally, in regard to the recently discovered CIM, due to its position next to the UBD, van den Heuvel et al. speculated that CSB‑CSA interaction is stabilized by binding of the UBD of CSB to auto-ubiquitylated CSA [39].

A comprehensive study by Lake et al. has highlighted the existence of an auto-regulatory mechanism of CSB protein function that involves all three regions of CSB [40]. In particular, mutational studies have shown that the central region of CSB displaying the ATPase activity and the C-terminal region are necessary for the stable binding of CSB to chromatin after exposure to UV radiation [40]. On the other hand, the N-terminal region of CSB is responsible for the specificity of the C-terminal binding to chromatin and acts as a negative regulator of this binding under normal conditions [40]. In response to genotoxic stress, the negative regulation of the N-terminal is alleviated, a process fueled by energy released from the hydrolysis of ATP [40]. Interestingly, in another study involving oxidative DNA lesions, angelicin mono-adducts or trioxsalen interstrand crosslinks (ICLs), a functional ATPase domain was not required for chromatin association [41]. In contrast, other studies have proven that the ATPase activity is essential for the assembly of NER factors and for loading and binding of homologous recombination (HR) proteins, while it is dispensable for processing of 8-oxoguanine, an oxidative base lesion [42,43,44]. The ATPase activity of CSB may also be affected by a high dose of trans-4-hydroxy-2-nonenal (HNE), one of the major lipid peroxidation products, and at the same time it has been shown that mutation of different ATPase motifs leads to different sensitivities to HNE [45]. The above observations suggest that the function of the structural elements of CSB is differentially regulated, depending on the type of damage, and therefore the type of repair mechanism activated. Another example of the differential regulation of CSB upon UV damage and in DSB repair is the fact that the first 30 amino acids reported as essential for HR-mediated repair of DSBs are dispensable for UV repair [19,38].

### 3.2. Post-Translational Modifications

Although structural regulation of a protein through folding and refolding plays a critical role in protein function, special reference should be made to its regulation via post-translational modifications. In effect, the modification of amino acids and of their side chains contributes significantly to the functional diversity of proteins and therefore may alter their activity, the balance between their expression and degradation and interactions with other proteins [48]. Especially for DNA repair proteins, such as CSB, their differential regulation via post-translational modifications is very important, as the alternative would be to synthesize the protein de novo, with the risk of synthesizing a mutated protein due to existing lesions. In the case of CSB, several studies have been conducted to identify its post-translational modifications, such as phosphorylation, ubiquitination, poly-ADP-ribosylation and SUMOylation, and their biological importance (Figure 1).

#### 3.2.1. CSB Phosphorylation

Regarding CSB phosphorylation, it has been found that upon exposure of cells to hydrogen peroxide, the CSB protein is phosphorylated by c-Abl kinase at tyrosine 932 [49]. As a result, the protein is redistributed in the nucleus and is enriched in the nucleolus. This altered subcellular localization of the phosphorylated CSB in response to oxidative stress suggests that phosphorylation of CSB by c-Abl may play an important role in the repair of oxidative damage [49]. In addition, Christiansen et al. suggest that CSB is phosphorylated under normal conditions and is dephosphorylated after exposure to UV irradiation, a modification which increased the ATPase activity of the protein [50]. In a similar manner, CSB has been found to be dephosphorylated in cells treated with HNE [45]. Moreover, damage-induced phosphorylation of CSB on S10 by ATM and cell cycle-dependent phosphorylation of CSB on S158 by cyclin A-Cdk2 was found to be essential for its chromatin remodeling activity at DSBs [19]. These phosphorylation events, which were found to be dispensable for the repair of UV-induced DNA lesions, are proposed to be responsible for the release of the auto-inhibitory signal of the N-terminal region on its ATPase domain [19,38]. Finally, mass spectrometry studies have identified several other potential phosphorylation sites of the CSB protein. Based on an in silico analysis, 29 potential phosphorylation sites were obtained using only proteomic discovery mass spectrometry [51]. The most prominent of these sites are the serine residues located at the N-terminus of the CSB protein at positions 158, 429, 430, 486 and 489 (Figure 1) [51].

#### 3.2.2. CSB Ubiquitination

**Figure 2:**
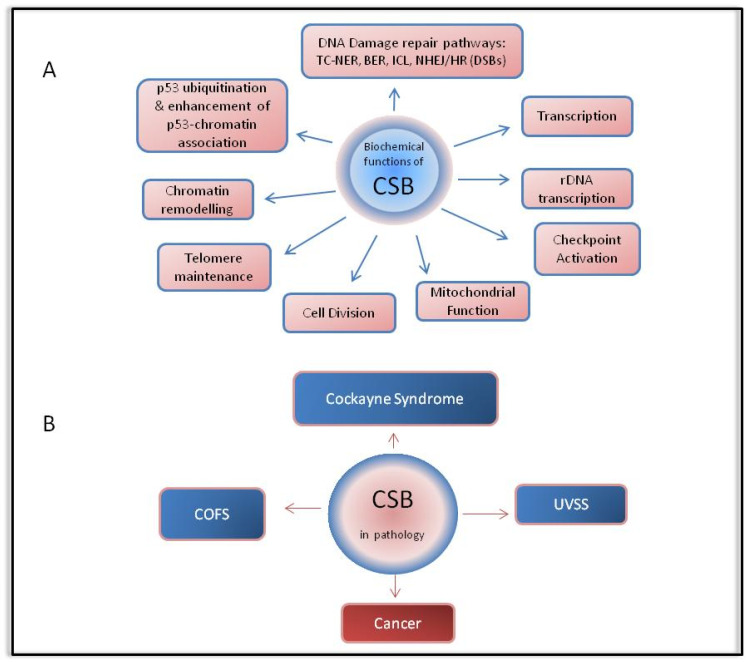
Multifunctional role of CSB (**A**) and related pathologies (**B**). Abbreviations: TC-NER: Transcription-Coupled Nucleotide Excision Repair, BER: Base Excision Repair, NHEJ: Non-Homologous End Joining, HR: Homologous Recombination, DSBs: Double-Strand Breaks, COFS: Cerebro-Oculo-Facio-Skeletal Syndrome, UVSS: UV-Sensitive Syndrome.

Several links between CSB and ubiquitin have been documented. First, as already mentioned, a small part of the carboxyl terminal of the CSB protein (less than 30 amino acids long), the so called UBD, has been identified as responsible for binding to ubiquitin (Figure 1) [32]. This region is regarded as essential for DNA repair via the TC-NER mechanism, since its deletion resulted in lower rates of excision and removal of lesions [32]. This notion was challenged by Takahashi et al., who identified the WHD, which folds as a single globular domain and interacts with ubiquitin via its second α-helix and C-terminal extremity, as the minimal ubiquitin binding domain of CSB [52]. Second, Groisman et al. identified CSB as a substrate of CSA for ubiquitination and degradation at the late stages of repair after UV irradiation [53]. On the other hand, Wei et al. identified a CSA-independent CSB protein ubiquitination pathway in which CSB is poly-ubiquitinated by the BRCA1 protein soon after exposure to UV irradiation, and even before repair by TC-NER is completed [54]. In addition, a site of CSB ubiquitination (at lysine residue 991) has been identified [55], acting as another example of differential regulation of the role of CSB in the different pathways, as it was found to be dispensable for TC-NER but was essential for repair of oxidative damage via the BER mechanism and genome stability [55]. Finally, a CSB deubiquitinating enzyme called Ubiquitin -specific protease 7 (USP7), together with its partner protein UVSSA, was identified and its role in increasing the protein levels after its initial decrease (an identified biphasic response) upon UV-induced DNA damage has been proposed in order to fine-tune TC-NER (Figure 1 and Figure 2) [56,57]. 

#### 3.2.3. CSB Poly-ADP-Ribosylation

Another post-translational modification of CSB, which takes place upon damage, is poly-ADP-ribosylation. In particular, it has been found that after exposure to oxidative stress, CSB is modified by poly (ADP-ribose) polymerase-1 (PARP1) enzyme and the addition of an ADP ribose and this modification results in the inhibition of CSB’s DNA-dependent ATPase activity [58]. The role of this inhibition still remains elusive. The authors speculate that this inhibition might be a secondary effect caused by an alteration in DNA binding of the modified CSB or that, since ATP hydrolysis by CSB has been shown to cause unwrapping of the DNA, this modification of CSB might result in an increase in DNA wrapping by CSB [19,58].

#### 3.2.4. CSB SUMOylation

Finally, the most recently identified post-translational modification of CSB, in response to UV irradiation, is SUMOylation (Figure 1). In one study, three potential SUMOylation sites were proposed, two at the carboxyl terminal (K1487, K1489) and one at the N-terminal of the protein (K205). However, both a double mutant CSBK1487R, K1489R (2K→R) as well as a triple mutant CSBK1457R, K1487R, K1489R did not abolish the modification of CSB by SUMO2 [28]. On the other hand, mutation of lysine 205 (K205) partially abolished SUMOylation of CSB and affected the function of CSB in TC-NER, resulting in the failure to recover RNA synthesis, which is a hallmark characteristic of CS cells, indicating a role for CSB SUMOylation in TC-NER [36,59]. In fact, very recently, Liebelt et al. targeted five lysines, which were embedded in the SUMO consensus motif (K32, K205, K481, K1359 and K1489) and after mutation analyses (including a K481, 1359 1489R triple mutant, a K205, 481, 1359 1489R quadruple mutant, a quintuple mutant and finally a K32, K205 double mutant) the authors concluded that CSB is SUMOylated predominantly at the two N-terminal lysines (32 and 205) [60]. Interestingly, the same study highlighted that active transcription and stalling of RNAPII at the site of DNA damage is a prerequisite for the modification of CSB by SUMO2. Furthermore, they show that the CSA–CRL4 complex regulates the stability of the modified protein in response to damage, and although the exact mechanism remains elusive, it does not involve a CSA-dependent ubiquitination and degradation of CSB after UV damage, as suggested by Groisman et al. [53,60].

## 4. CSB in Pathology

### 4.1. Cockayne Syndrome

Cockayne syndrome (CS) is characterized by a broad spectrum of clinical features including cachectic dwarfism, cutaneous photosensitivity, microcephaly, growth and developmental abnormalities, neurological and retinal degeneration, physical impairment, deafness and premature aging (reviewed in [61]). Analyses of large cohorts of CS patients have shown that there is no definite correlation between the genotype (mutations identified) and the symptomatology (clinical manifestations) [62,63]. In fact, as far as CSB mutations are concerned, neither the affected region nor the nature of the mutation is linked to specific clinical manifestations or to the severity of the disease, although a tendency to more severe phenotypes has been proposed in patients with mutations downstream of the PiggyBac insertion in intron 5 [62]. Interestingly, almost all of the missense mutations analyzed were positioned in or next to one of the seven helicase domains, a fact that underlines the clinical importance of these domains [62]. Apart from the two principal complementation groups of CS (CSA and CSB) with mutations in the *ERCC8* and *ERCC6* genes, respectively, a small number of CS cases have been reported to carry mutations in the *ERCC1* and *ERCC4* (xeroderma pigmentosum complementation group F-*XPF*) genes [64].

### 4.2. Models of Cockayne Syndrome

In order to decipher the systemic effects and to better understand the mechanisms of Cockayne syndrome progression, several animal models have been generated, including mice, *Caenorhabditis elegans*, zebrafish and, recently, rats. The first mouse model (CSB^m/m^), which recapitulated some of the CS phenotypic characteristics, was developed by introducing the same truncation mutation found in a human CS1AN patient [65]. The CSB-deficient mice exhibited similar characteristics to their human counterpart cell models, including UV sensitivity, deficient TC-NER, proficient global genome nucleotide excision repair (GG-NER or GGR, a subpathway of NER responsible for the repair of bulky DNA lesions throughout the genome) and inability to recover RNA synthesis after UV irradiation [65]. In addition, as far as their clinical manifestations are concerned, the mice exhibit photophobia, parakeratosis, minor growth disturbance, deafness and mild neurodegeneration [65]. However, in contrast to humans, they did not show signs of severe neurodegeneration, impaired sexual development or reduced lifespan [54]. Notably, the mutant mice, in contrast to human CS patients, appear to have increased susceptibility to cancer [65]. It is important to emphasize on a source of confusion in the literature concerning the fact that not only CS patients, but also XP patients, exhibit neurodegeneration [12,66]. However, XP neurologic disease is very different from CS neurodegeneration as it affects primarily the large neurons in many brain and spinal cord regions as well as in the peripheral nervous system [12,66]. On the other hand, in CS neurodegeneration, the myelin-forming glial cells (oligodendrocytes) are primarily affected and, as a result, tigroid demyelination is observed [12,66]. Moreover, CS patients develop calcifications in the basal ganglia and in the cerebellar white matter and may also develop brain vascular defects [12,66,67]. Although the single CSB knockout mouse model failed to accurately mimic the typical disease manifestation seen in humans, a double knockout mouse lacking both the *XPA* or *XPC* and the *CSB* genes produced a more CS-like phenotype, presenting severe neurodegeneration, compromised growth, low weight, premature death, etc. [68,69]. Furthermore, depletion of CSB by RNA interference in *C. elegans* (csb−1) led to hypersensitization to UV exposure and resulted in enhanced germ cell proliferation arrest and apoptosis and increased embryonic lethality, whereas depletion of CSB in zebrafish embryos using antisense morpholino oligonucleotides resulted in severe developmental abnormalities upon UV damage [70,71]. Finally, the first rat model of CS was recently developed using CRISPR/Cas9-mediated genome editing [72]. The Csb-deficient rats (CsbR571X) demonstrated brain abnormalities such as cerebellar atrophy, thinning of the layers of the cerebellar cortex and degeneration of Purkinje neurons, which were features that have already been seen in some CS mouse models (such as Xpg -/-), but they also displayed reduced myelination in the cerebellum, the key aforementioned notable feature of the human CS neurologic disease, a characteristic not seen in CSB mice [72,73].

### 4.3. Other CSB-Related Pathologies

Apart from Cockayne syndrome, mutations in the *ERCC6* gene (CSB) are implicated in the clinical manifestation of two other TC-NER deficiency syndromes, so-called UV-sensitive syndrome (UVSS) and cerebro-oculo-facio-skeletal (COFS) syndrome (Figure 2). Cells from UVSS patients are UV sensitive, are characterized by deficient TC-NER and exhibit almost identical cellular and biochemical responses to UV compared to CS patients [74]. On the other hand, UVSS patients share only mild symptoms with CS, such as photosensitivity, mild freckling and telangiectasia, and notably show no signs of neurological or growth abnormalities [75]. Spivak and Hanawalt proposed that the aforementioned difference lies in the fact that UVSS patients, in contrast to CS patients, are proficient in repair of oxidative base damage [76]. Another interesting fact, opposite from what one might expect, is that a mutation (R77X), which resulted in incomplete absence of a functional CSB (null mutation), did not cause a more severe phenotype with signs of developmental or/and neurological defects but instead was characterized only by the mild symptoms present in UVSS [77]. An explanation proposed by Horibata et al. is that in CSB cells, truncated CSB polypeptides are produced, which may interfere with the essential cellular processes of repair, transcription and transcriptional bypass or repair of oxidative DNA damage, resulting in a more severe clinical phenotype [77]. Apart from *ERCC6* mutations, UVSS is also caused by mutations in the *ERCC8* (CSA) and UV-stimulated scaffold protein A (*UVSSA*) genes. As mentioned above, UVSSA has been found to protect CSB from UV-induced degradation, by targeting the ubiquitin-specific protease USP7 to a DNA lesion-stalled RNAPII complexes [56,57].

COFS syndrome represents the most severe end of the CS spectrum and appears to have an early onset of symptoms [78]. Typical symptoms are congenital microcephaly, congenital cataracts and/or microphthalmia, arthrogryposis, severe developmental delay, severe postnatal growth failure and facial dysmorphism [79]. Similarly to CS and UVSS cells, cells derived from COFS syndrome patients are UV sensitive and TC-NER deficient [78]. Genes involved in the manifestation of the syndrome are *CSB, XPD (ERCC2), XPG (ERCC5)* and *ERCC1* [80,81,82,83].

Apart from UVSS and COFS syndrome, an inactivating mutation of *CSB* has been reported in two CSB siblings showing symptoms of DeSanctis‑Cacchione (DSC) syndrome, which is a rare and severe form of XP with severe neurological abnormalities. Interestingly, identical alterations have been reported in a patient with typical CS features, a fact that underlines the complexity of correlating the genetic background to specific phenotypes [84].

Finally, from the point of view of CS-related pathologies, the rare combined XP/CS phenotype, caused by specific mutations in *XPD (ERCC2), XPB (ERCC3), XPF (ERCC4)* or *XPG (ERCC5)* genes, should be analyzed [85]. XP/CS patients develop combined clinical feature of XP and CS syndrome, showing, on the one hand, increased cancer risk and skin sensitivity and, on the other hand, severe developmental abnormalities such as short stature, deficient sexual development and retinal atrophy similar to CS patients [1].

### 4.4. The Role of CSB in Cancer

Impaired NER has been associated with an increased prevalence of neurodegeneration and cancer. On the one hand, CS patients are characterized by neurological abnormalities and, although photosensitive, do not develop cancer [86,87,88]. On the other hand, XP patients are 1000 times more prone to developing cancer [89]. Reid-Bayliss et al. suggested that this increased susceptibility is due to the fact that CS cells, in contrast to XP cells, do not show increased levels of UV-induced mutagenesis [89]. Notably, Caputo et al. have shown that CSB is overexpressed in a number of cancer cell lines from different tissues and acts as an anti-apoptotic factor for cancer cells, tipping the balance towards cell proliferation and survival, and away from cell cycle arrest and senescence [90]. Therefore, it is postulated that a lack of carcinogenicity seen in CS patients is a derivative of increased apoptosis of DNA-damaged cells and cellular growth inhibition [90].

Overexpression of CSB in cancer cells supports the notion that CSB also plays an important role in cancer development (Figure 2). In fact, it seems that CSB displays a multifunctional role in this context as well [91]. Firstly, accumulation of the tumor suppressor p53 results in either increased levels of apoptosis or growth arrest [92,93,94]. Inversely, p53 inactivation promotes not only the initiation of tumorigenesis, but also possible metastasis, recurrence and lethality [93,94,95,96]. It is therefore notable that CSB is part of an E3 ubiquitin ligase complex together with CSA, Mdm2 and p53, and controls p53 levels by targeting it for ubiquitination in an Mdm2-dependent manner [97]. In line with these findings, Paccosi and Proietti-De-Santis recently proposed a model in which the sequestration of CS proteins to the site of damage may act as a “biological dosimeter” to modulate the activity of p53 and therefore cell fate [98]. Furthermore, CSB has been proven to act as a mediator of the hypoxic response by redistributing the transcriptional co-activator p300 between hypoxia-inducible factor 1 (HIF1) and p53 [99]. Tolerance to hypoxia (limited supply of oxygen), is a prominent characteristic that cancer cells have developed in order to survive in a situation during which the pre-existent vascularization cannot support their increasing mass [93,100]. HIF1 activates the transcription of pro-survival genes implicated in angiogenesis, such as VEGF, and anaerobic glycolysis, such as GAPDH, and therefore plays a major role in enabling cancer progression [95].

In addition to hypoxia adaptation, cancer cells need to respond to other type of stress conditions, such as oxidative stress, for which balance of the intracellular reactive oxygen species (ROS) levels is required, and endoplasmic reticulum (ER) stress, which triggers the unfolded protein response (UPR) (reviewed in [93,101,102]). As far as the former is concerned, CSB appears to be involved in the control of the cellular redox balance and repair of oxidative DNA lesions in the nucleus and in mitochondria and appears to act as an electron scavenger in the mitochondria [103,104,105]. In regard to ER stress, CSB seems to limit the level of misfolded proteins, while its suppression results in upregulation of pro-apoptotic factors of the UPR-mediated apoptosis pathway and downregulation of the UPR pro-survival mediators [106].

## 5. Future Prospects—Potential Therapeutic Targeting of CSB

Given the multiple roles CSB may play in cancer progression, a challenging task is whether CSB can be an attractive candidate for therapeutic targeting. First and foremost, it should be noted that ablation of CSB by antisense technology not only resulted in increased levels of apoptotic death of cancer cells but most importantly did not affect the normal cells, a key prerequisite for any candidate therapeutic approach [90].

Moreover, considering the role that CSB has in transcription-coupled repair of bulky DNA adducts produced by platinum-based chemotherapeutic agents used in cancer therapy, and the fact that the silencing of CSB by RNA interference has been proven to increase the sensitivity of tumor cells to the chemotherapeutic agent cisplatin, one can acknowledge the important effect that the silencing of CSB may confer in minimizing the chemotherapeutic dose required to induce apoptosis, thereby reducing chemotherapy side effects [91,107].

There are several *ERCC6* SNPs (Single-nucleotide polymorphism) that have been associated with increased cancer susceptibility or affected the response to chemotherapy. Such examples include rs3793784: C > G (NC_000010.11:g.49539493G > C), an *ERCC6* variant, which alters its transcriptional activity and may increase lung cancer susceptibility, as well as rs4253002: G > A (NC_000010.11:g.49539292C > T) and rs4253212: G > A (NC_000010.11:g.49470166G > A), SNPs which are associated with toxicities (gastrointestinal toxicity and neutropenia, respectively) after platinum-based chemotherapy in patients with advanced non-small cell lung cancer [108,109]. On the other hand, there are SNPs, such as rs12571445 (NC_000010.11:g.49514137A > G) and rs2281793 (NC_000010.11:g.49519496C > T), which are associated with progression-free survival and overall survival, respectively, after platinum-based chemotherapy in patients with advanced non-small cell lung cancer [108,109]. Additionally, a specific *ERCC6*-Q524* (COSV63389787; NP_000115.1: c.1570C > T) mutation has been found to increase cisplatin sensitivity of epithelial ovarian cancer cells in vitro [110]. Finally, a study, in which 193 DNA repair genes were evaluated in regard to their mutation frequency in sequenced tumor samples from the COSMIC database, revealed that *ERCC6* is among the top 20 most frequently mutated genes in lung, breast and skin cancers [111]. Therefore, an analysis of the different polymorphisms may result in a number of interesting candidate gene loci to be further evaluated for therapeutic targeting.

In conclusion, considering the multiple roles CSB may play in cancer progression, it appears that inactivation of specific CSB loci, in a personalized manner, may significantly contribute to cancer therapy.

## Data Availability

Not applicable.
